# Phosphorylation of ovalbumin after pulsed electric fields pretreatment: Effects on conformation and immunoglobulin G/immunoglobulin E-binding ability

**DOI:** 10.3389/fnut.2022.932428

**Published:** 2022-08-12

**Authors:** Wenhua Yang, Wenjing Duan, Qiuhong Li, Dengle Duan, Qin Wang

**Affiliations:** ^1^School of Chemical and Biological Engineering, Yichun University, Yichun, China; ^2^Guangdong Provincial Key Laboratory of Lingnan Specialty Food Science and Technology, Zhongkai University of Agriculture and Engineering, Guangzhou, China

**Keywords:** pulsed electric fields, phosphorylation, ovalbumin, structure, IgE-binding, IgG-binding, egg allergy

## Abstract

Ovalbumin (OVA) is one of major allergens of hen egg white with excellent nutritional and processing properties. Previous research exhibits that pulsed electric field (PEF) treatment could partially unfold OVA. This may contribute to the improvement of OVA phosphorylation. In this study, the effect of PEF pretreatment combined with phosphorylation on the structure and immunoglobulin (Ig) G/IgE-binding ability of OVA was investigated. The structural changes were measured by circular dichroism (CD), ultraviolet absorption, and fluorescence spectroscopy. The IgG- and IgE-binding abilities were determined by inhibition enzyme-linked immunosorbent assay (ELISA) using rabbit polyclonal antibodies and egg-allergy patients’ sera, respectively. The results showed that PEF pretreatment combined with phosphorylation markedly reduced the IgG- and IgE-binding abilities. It was attributed to the changes in secondary and tertiary structure, which was reflected in the increase of ultraviolet (UV) absorbance, α-helix content, and the increase the molecular weight. Moreover, it suggested PEF pretreatment improved the phosphorylation of OVA and enhanced the reduction of IgG/IgE-binding capacity of phosphorylated OVA. Therefore, PEF pretreatment combined with phosphorylation has the potential for developing a method for OVA desensitization.

## Introduction

Hen’s egg is one of the most important daily nutrition resources and food ingredients. It has been widely applied into the food industry because of its outstanding nutritional and functional properties, such as excellent absorption, gelation, and emulsification (1). However, it causes 1–3% of food allergies in children and ranks among the top allergic foods in Europe (2). For patients with egg allergy, the most effective measure to prevent egg allergy is completely avoiding egg components, often very difficult due to the widespread use of egg proteins in the food industry (3). Therefore, there is considerable interest in developing a safe and effective method for decreasing the allergenicity of hen’s eggs.

Ovalbumin (OVA), named as Gal d 2, which constitutes about 54% of egg white proteins, is a 45-kDa glycoprotein with 385 amino acids. It becomes an ideal experimental model protein of food allergy because of its strong allergenicity and available quantity (4). Numerous processing methods have been investigated to change allergenicity of OVA, such as heating (5), γ-irradiation (6), pulsed electric field (PEF) (7), ultrasound treatment (8), glycation (9), enzymatic hydrolysis under high pressure (10), and ultrasound combined with glycation (4, 11). However, it is still unclear how these processing, even more those based on novel technology, affect the allergenicity of OVA (12). Among them, the combined method has a good effect on reducing OVA allergenicity and becomes a hot spot of research on OVA desensitization.

Phosphorylation is a common protein modification that realized by chemical or enzymatic method (13). It is generally recognized as an effective method to improve protein functional properties, namely, solubility, emulsification, and thermal stability (14). For the enzymatic phosphorylation, amino acid residues such as serine, tyrosine, and threonine in food protein could be phosphorylated by protein kinases through covalent bonds with phosphate groups. It is reported that there are two phosphorylation sites of S68 and S344 in OVA during post-translational modification, making OVA have three forms with no, one and two phosphates (15). For the chemical phosphorylation, phosphorylation site is more than just the hydroxyl amino acids. Xiong et al. (16) found that the serine, threonine, lysine, and arginine residues were bound to phosphate groups with covalent interactions between OVA and sodium tripolyphosphate (STPP). Furthermore, Sheng et al. (17) revealed other phosphate sites (S221, S269, Y281, and S324) in OVA that phosphorylated under wet-heating by using liquid chromatography with mass spectrometry. They proved that phosphorylation dramatically improved the emulsifying and foaming properties of OVA. Besides, Costa et al. (12) showed that phosphorylated OVA has higher immunoglobulin (Ig) E-binding capacity compared with its dephosphorylated forms. Therefore, phosphorylation has the potential for modifying functional properties of OVA.

Compared with the enzymatic phosphorylation, chemical phosphorylation has some advantages, such as low cost and significant phosphorylation effect. It could not only improve some of OVA functional properties, but also could promote calcium absorption by using as a dietary supplement because of the increase in the solubility of calcium phosphate through introduction of phosphate groups (18). Recently, a number of studies are conducted to investigate the effect of phosphorylation on the functional properties of OVA. Most of them focus on the heat stability, emulsifying, and gelling properties of OVA (16–20). However, there is few studies of phosphorylation on the allergenicity of OVA. Moreover, single phosphorylation treatment of OVA could not achieve the desired effect because of the hiddenness of some phosphorylation sites at the nature status. Thus, it is necessary to explore a combined processing method to promote phosphorylation of OVA.

Pulsed electric field (PEF) is an emerging promising non-thermal technology for food processing and preservation (21). Compared with the conventional thermal processing methods, it could keep the food quality attributes that maybe lost during conventional thermal processing (22). Extensive literatures show that PEF has been applied to food sterilization, enzyme inactivation, intracellular components extraction, and pesticide degradation (23). In the previous work, we found PEF at low-electric field intensity (below 25 kV/cm, for 180 μs) or for short time (less than 60 μs, at 35 kV/cm) could increase the IgG- and IgE-binding capacities due to the partial unfolding of OVA (7). Moreover, PEF could improve glycation of β-lactoglobulin through exposure of glycation site by partial unfolding β-lactoglobulin (24). It may allow more efficient phosphorylation of OVA. Therefore, the combination of PEF and phosphorylation is worth applying to protein modification. However, limited comprehensive research has been focused on the influence of PEF pretreatment combining with phosphorylation on immunogenic and structural properties of OVA.

In the present study, phosphorylation combined with PEFs pretreatment was used to treat OVA, aiming to evaluate the effect of phosphorylation after PEFs pretreatment on IgG/IgE-binding ability and structural properties of OVA, and also to expound upon their relationships. It will enrich the relevant theory of chemical phosphorylation combined with the PEFs for OVA desensitization.

## Materials and methods

### Materials

Goat anti-rabbit IgG–HRP conjugate, goat anti-human IgE–HRP conjugate, STPP and 1-anilinonaphthalene-8-sulfonate (ANS) were purchased from Sigma-Aldrich (St. Louis, MO, United States). Egg allergy patients’ sera were bought from PlasmaLab International (Everett, WA, United States) and frozen (–80°C) until analysis. Their specific IgE levels ranged from 10.8 to 64.6 kU/L (25). The polyclonal antisera OVA were produced from four Japanese male rabbits. All the chemicals used were of analytical grade.

### Sample preparation

Ovalbumin was isolated and purified from fresh hen eggs by using ammonium sulfate precipitation, isoelectric precipitation, and ion exchange chromatography (8), and its purity was more than 98%. To reduce the thermal effect during PEF pretreatment, the electrical conductivity of sample solution was maintained below 0.1 S/m. Then, 1 g of OVA was dissolved in 100 ml of 10 mmol/L phosphate buffer (pH 8.0) and treated by a bench scale pulse generator system with a unipolar square wave (designed by the Tsinghua University, Beijing, China). There were two co-field flow treatment chambers with the gap distance and inner diameter of 0.4 cm in PEF-processing system. The temperature was real-time monitored and kept below 20°C by ice bath. After processing, the STPP was added into the solutions to final concentration of 0.1 mol/L. Then, it was freeze-dried and incubated at 50°C and 79% relative humidity for 2 h. After phosphorylation, the remaining phosphates were removed by an ultrafilter (3,000 Da cutoff; Millipore, Billerica, MA, United States). Then, all the phosphorylated samples were adjusted to 1 mg/ml and stored at 4°C for less than 48 h. Untreated OVA was used as control and named OVA-N. OVA heated at phosphorylation conditions without phosphates was used as another control and named OVA-H. The phosphorylated samples PEF-pretreated for 180 μs at 0, 20, 25, and 30 kV/cm were named OVA-P, OVA-P-20, OVA-P-25, and OVA-P-30, respectively. PEF-treating time (*t*) was calculated as follows (26):


(1) $$t=\frac{n\times V\times f\times W}{v}$$

where *n* is the number of treatment chambers, *V* is the volume of a chamber (ml), *f* is the pulse repetition rate (pulses per second, Hz), *W* is the pulse width (μs), and *v* the flow rate (ml/s). In this study, the pulse repetition rate and pulse width were 20 Hz and 5 μs, respectively. The flow rate of OVA sample was 0.33 ml/s.

### Determination of immunoglobulin G- and immunoglobulin E-binding capacities

The IgG- and IgE-binding capacities of OVA were determined by inhibition enzyme-linked immunosorbent assay (ELISA) with rabbit polyclonal anti-OVA-sera and egg allergy patients’ sera, respectively (27). At first, a 96-well microplate was coated with 100 μl of 2 μg/ml native OVA (dissolved in 0.05 mol/L PBS pH 7.4) and incubated overnight at 4°C. Then, it was blocked with 50 mg/ml skimmed milk for 1 h at 37°C. Next, 50 μl of either pooled rabbit antisera (diluted to 1:12,800) or pooled human sera (diluted to 1:8) and serial diluted OVA samples (inhibitors, 0.5–60 μg/ml) were added and incubated at 37°C for 30 min. Subsequently, 100 μl of goat anti-rabbit IgG–HRP conjugate (diluted to 1:5,000) or goat anti-human IgE–HRP conjugate (diluted to 1:5,000) was added and incubated for 30 min at 37°C. The microplate was washed for five times with PBST (0.05% Tween-20 in PBS) at every step aforementioned. Then, 100 μl fresh prepared TMB solution that contained 166 mM sodium acetate, 13 mM citric acid, 0.009% hydrogen peroxide, 0.6 mM ethylenediaminetetraacetic acid disodium salt, 6.2 mM TMB, and 5% glycerol was added and incubated for 15 min at 37°C. Finally, 50 μl of 2 mol/L sulfuric acid was added to stop the reaction and the absorbance was measured at 450 nm using a microplate reader (HF2000, Huaan Magnech, Beijing, China). The inhibition rate was calculated as follows: inhibition = (1 – B/B_0_) × 100%, where B and B_0_ are the absorbance values of the well with and without the inhibitor, respectively. IC_50_ is the concentration of inhibitors that causes a 50% inhibition of antibody binding (μg/ml) (25).

### Marix-assisted laser desorption ionization time-of-flight mass spectrometry analysis

According to the method of Zhang et al. (28), with some modifications, the molecular weights of OVA samples were determined by using a 4800 MALDI–TOF/TOF mass spectrometer (AB Sciex, Framingham, MA, United States) with the positive ion mode. The diluted samples (0.1 mg/ml) were mixed 1:1 with the matrix (10 mg/ml sinapic acid in 50% acetonitrile with 0.1% TFA), and then 1.0 μl mixture was spotted onto the MALDI target and air-dried before analysis. The mass spectrum of each sample that was spotted on at least three individual target positions was averaged from 80 individual spectra.

The average number of phosphate molecules attached to each OVA molecule, evaluated as incorporation ratio (IR), was used to assess the degree of phosphorylation (29). Based on the molecular weight (MW) shift from native OVA to phosphorylated OVA, the IR of phosphate to OVA could be deduced as:


(2) $$\mathrm{IR}=\left({\text{MW}}_{\text{PA}}-{\text{MW}}_{\text{NA}}\right)/79.9799$$

where 79.9799 is the molecular weight of phosphates attached to OVA, MW_*PA*_, and MW_*NA*_ are the molecular weight of phosphorylated OVA and native OVA, respectively.

### Far-ultraviolet circular dichroism spectroscopy

The secondary structure of OVA was defined by far-ultraviolet (UV) (190–240 nm) circular dichroism (CD) spectroscopy. The MOS-450 spectropolarimeter (Bio-Logic SAS, Claix, France) was used to collect the CD spectra of OVA (0.1 mg/ml) at scan speed of 100 nm/min. The path length, bandwidth and step resolution were all 0.1 cm. The data were showed as molar residue ellipticity [(θ), deg cm^2^/dmol], and the contents of different secondary structures were analyzed with DichroWeb.^1^

### Ultraviolet absorption and intrinsic fluorescence spectroscopy

The UV-2910 Hitachi spectrophotometer (Hitachi, Tokyo, Japan) was applied to collect the UV absorption spectra. The OVA samples (0.5 mg/ml) were scanned from 240 to 320 nm at a scan speed of 800 nm/min. The F-7000 Hitachi spectrofluorometer (Hitachi, Tokyo, Japan) was used to measure the intrinsic fluorescence of OVA (0.2 mg/ml). The emission spectra were scanned from 300 to 420 nm at a speed of 1,200 nm/min with the excitation wavelength of 280 nm. The bandwidth, excitation, and emission slits were all 5.0 nm.

### Surface hydrophobicity analysis

According to the method of Yang et al. (24), with modification, ANS fluorescence was used to measure the surface hydrophobicity of OVA. The volume ratio of sample (0.25–1.0 mg/ml) and ANS solution (8 mmol/L) was 200:1. The relative fluorescence intensity was determined by using an F-7000 Hitachi spectrofluorometer (Hitachi, Tokyo, Japan). The excitation wavelength was 390 nm and the emission spectrum was scanned from 400 to 600 nm at a speed of 1,200 nm/min. The bandwidth, excitation, and emission slits were all 5.0 nm. The surface hydrophobicity (H_0_) was the initial slope of the fluorescence intensity vs. protein concentration plot, which was calculated by linear regression analysis.

### Statistical analysis

All the experiments were carried out in triplicate, and all results were presented as mean value ± SD. The statistical analysis was performed by ANOVA with SPSS 19.0 (SPSS Inc., Chicago, IL, United States) and OriginPro 2020 (OriginLab Corp., Northampton, MA, United States).

## Results

### Immunoglobulin G- and immunoglobulin E-binding capacities analysis

**FIGURE 1:**
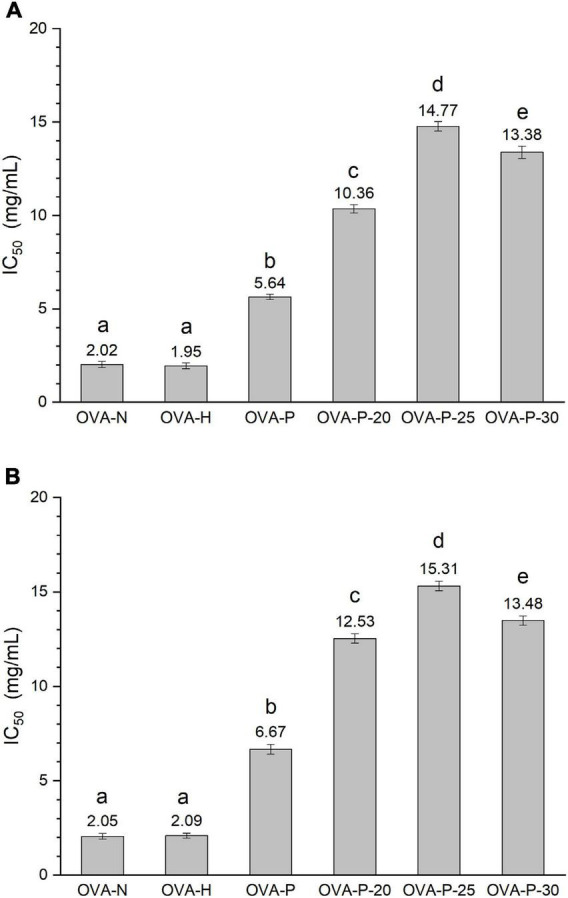
Changes in the immunoglobulin (Ig) G **(A)** and IgE **(B)** binding abilities of ovalbumin (OVA) samples, which were determined by inhibition enzyme-linked immunosorbent assay (ELISA). IC_50_ value is the inhibitor concentration that causes a 50% inhibition of the antibody-binding capacity. Different letters (a–e) on the top of the bars denote significant difference (*p* < 0.05).

In this work, the IgG- and IgE-binding abilities of OVA samples were determined by inhibition ELISA with rabbit polyclonal anti-OVA-sera and egg-allergy patients’ sera, respectively. The IC_50_ value is the inhibitor concentration that causes a 50% inhibition of the antibody-binding capacity, which reflecting that the higher the IC_50_ value, the lower the binding capacity. Figure 1 shows the changes of IC_50_ values of OVA treated under different conditions. As shown in Figure 1A, the IC_50_ value of OVA-H was almost the same as that of OVA-N. However, after OVA was phosphorylated with phosphates, it markedly increased from 2.02 mg/ml (OVA-N) to 5.64 mg/ml (OVA-P). Moreover, when phosphorylated with PEF pretreatment, OVA showed further higher IC_50_ values, with the highest value (14.77 mg/ml) observed at PEF pretreatment at 25 kV/cm. Similar results were shown in Figure 1B. There was no significant difference between OVA-N and OVA-H (*p* < 0.05). However, OVA-P had higher IC_50_ value than OVA-N. Furthermore, phosphorylated OVA with PEF pretreatment had further higher IC_50_ values and OVA-P-25 had the lowest IgE-binding ability (15.31 mg/ml).

### Molecular weight analysis

**FIGURE 2:**
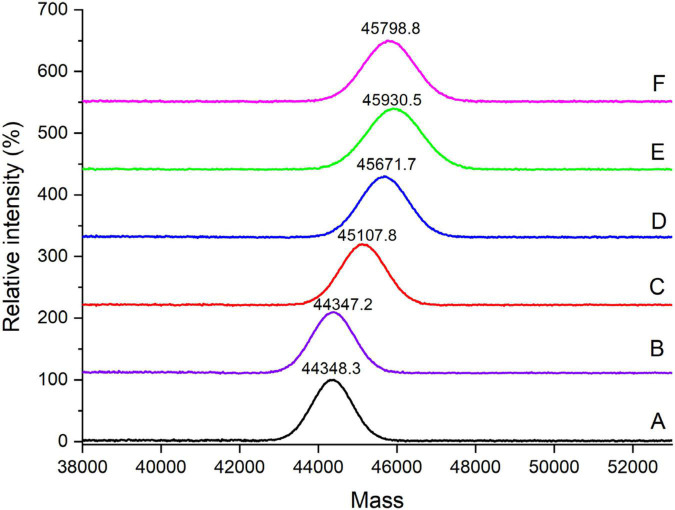
MALDI–TOF mass spectra of ovalbumin (OVA) samples. A–F represents OVA-N, OVA-H, OVA-P, OVA-P-20, OVA-P-25, and OVA-P-30, respectively.

Phosphorylation is the result of the typically covalent binding of the phosphates to proteins, without participation of enzyme. It will result in the increase of the molecular weight of protein. Figure 2 shows the changes in MALDI–TOF mass spectra of the OVA samples. There was no significant difference of molecular weight between OVA-N and OVA-H, indicating that dry-heating without phosphates did not change the molecular weight of OVA. However, the molecular weight of phosphorylated OVA samples (OVA-P, OVA-P-20, OVA-P-25, and OVA-P-30) increased from 44348.3 (OVA-N) to 45107.8, 45671.7, 45930.5, and 45798.8 Da, respectively. Moreover, the IR of OVA-P was 9.5, whereas that of OVA-P-20, OVA-P-25, and OVA-P-30 were 16.5, 19.7, and 18.1, respectively. Besides, the peak width of phosphorylated OVA after PEF pretreatment were much broader than that of phosphorylated OVA without PEF pretreatment because of the greater IR.

### Secondary structure analysis

**FIGURE 3:**
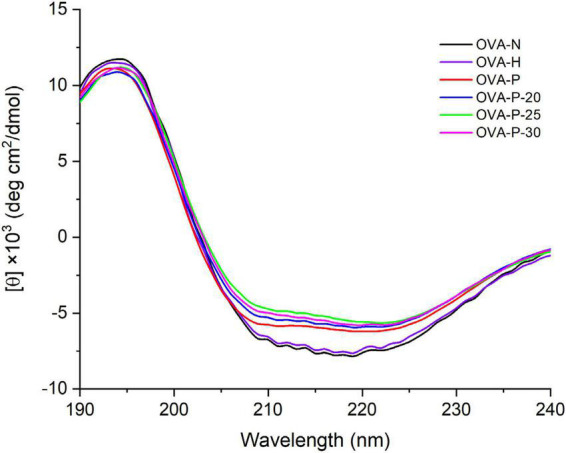
Changes in far-ultraviolet (UV) circular dichroism (CD) spectra of the ovalbumin (OVA) samples.

The changes of the far-UV CD spectra of OVA samples were depicted in Figure 3, which was consistent with the secondary structure content shown in Table 1. There were no significant changes (*p* < 0.05) in CD spectra of OVA after dry-heating without phosphates. However, the α-helix content of OVA phosphorylated with STPP increased from 24.1% (OVA-N) to 28.5% (OVA-P) while the β-sheet content decreased from 25.5% (OVA-N) to 19.8% (OVA-P). Furthermore, compared with OVA-P, the phosphorylated OVA with PEF pretreatment had more α-helix content and less β-sheet content. Besides, the unordered content of phosphorylated OVA increased by up to 10.1% when it is compared with that of OVA-N.

**TABLE 1:** The secondary structure contents (%) of the ovalbumin (OVA) samples.

Samples	α -Helix	β -Strand	Turn	Unordered
OVA-N	24.1 ± 0.2^a^	25.5 ± 0.3^a^	21.7 ± 0.3^a^	28.7 ± 0.2^a^
OVA-H	24.7 ± 0.3^a^	26.2 ± 0.5^a^	21.2 ± 0.2^a^	27.9 ± 0.4^a^
OVA-P	28.5 ± 0.4^b^	19.8 ± 0.3^b^	21.7 ± 0.6^a^	30.0 ± 0.2^b^
OVA-P-20	29.4 ± 0.5^b,c^	17.5 ± 0.6^c^	21.5 ± 0.7^a^	31.6 ± 0.5^c^
OVA-P-25	31.5 ± 0.3^d^	16.5 ± 0.4^c^	21.9 ± 0.8^a^	30.1 ± 0.4^b^
OVA-P-30	30.3 ± 0.7^c,d^	18.7 ± 0.4^c,b^	21.6 ± 0.6^a^	29.4 ± 0.7^a,b^

Values followed by different letters (a–d) in the same column are significantly different (*p* < 0.05).

### Ultraviolet absorption and intrinsic fluorescence spectra analysis

**FIGURE 4:**
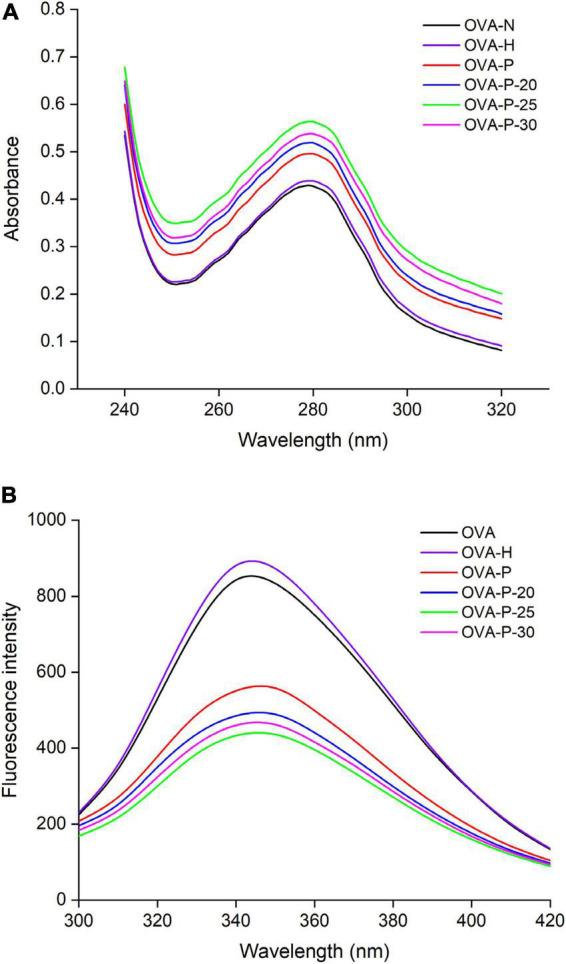
Changes in ultraviolet (UV) absorption **(A)** and intrinsic fluorescence spectra **(B)** of the ovalbumin (OVA) samples.

Figure 4A shows the changes of the UV absorption spectra of OVA samples. Compared with the native OVA, OVA dry-heated without phosphates had insignificant increase in UV absorbance intensity at 280 nm. After phosphorylation with phosphates, however, OVA displayed much higher UV absorbance intensity at 280 nm than native OVA. Moreover, phosphorylated OVA with PEF pretreated had higher value than that without PEF pretreatment, with the highest value observed at PEF intensity of 25 kV/cm (OVA-P-25).

The changes in the intrinsic fluorescence spectra of OVA samples were shown in Figure 4B. The maximum fluorescence emission (λ_*max*_) intensity of OVA slightly increased from 843 (OVA-N) to 881 (OVA-H) after dry-heated without phosphates. The result was consistent with UV absorption analysis, suggesting that dry-heating at 55°C for 4 h had limited influence on the OVA tertiary structure. However, after phosphorylation, the λ_*max*_ intensity of OVA-P, OVA-P-20, OVA-P-25, and OVA-P-30 decreased to 551, 485, 433, and 460, respectively.

### Surface hydrophobicity analysis

**FIGURE 5:**
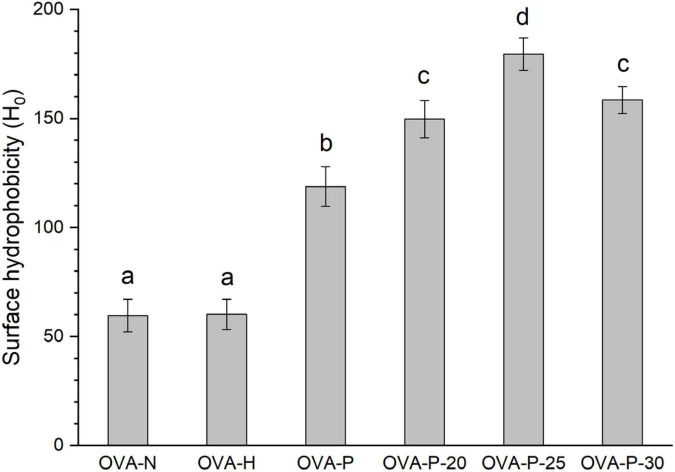
Changes in surface hydrophobicity of the ovalbumin (OVA) samples. Different letters (a–d) on the top of the bars denote significant difference (*p* < 0.05).

Figure 5 depicted the changes in surface hydrophobicity of OVA samples. There was no significant difference (*p* < 0.05) in surface hydrophobicity of OVA after dry-heating without phosphates, implying that dry-heating had little effect on the surface hydrophobicity of OVA. However, after phosphorylation with phosphates, the surface hydrophobicity of OVA dramatically increased from 59.6 (OVA-N) to 118.8 (OVA-P). Moreover, when OVA was phosphorylated after PEF pretreatment, its surface hydrophobicity was further decreased, with the highest value of 179.5 (OVA-P-25).

## Discussion

Phosphorylation, which is one of the common modifications in proteins, influences the functional properties of proteins *via* a covalent bond between proteins and phosphates. According to the ways of phosphorylation, it is divided into enzymatic phosphorylation and chemical phosphorylation. The latter has more application potential than the former due to its low cost and convenience. Phosphorylation has been used to improve protein functional properties among them allergenicity (30). However, the reduction extent of allergenicity is insufficient because of the limited phosphorylation degree. Our previous work showed that PEF could make OVA molecule partially unfold, which may contribute to the improvement of phosphorylation (7). Furthermore, STPP has good phosphorylation extent and dry-heating phosphorylation is more effective than wet-heating phosphorylation (16, 20). Therefore, OVA was phosphorylated with STPP under dry-heating condition, and the influence of phosphorylation combined with PEF pretreatment on the structure and IgG/IgE-binding ability of OVA was studied in this work. The results showed that phosphorylation combined with PEF pretreatment remarkably decreased the IgG- and IgE-binding abilities, which reflected in the increase of IC_50_ values were closely related to the structural changes of OVA. It is hypothesized that the masking of linear epitopes caused by the modification of acid amino residues dominantly accounts for the decreased IgG- and IgE-binding capacities of OVA, and the destruction of conformational epitopes that caused by structural changes subordinately resulted in the reduction.

The increase in IR of OVA clearly proved that phosphorylation increased the molecular weight of OVA, and PEF pretreatment significantly promoted the phosphorylation degree and led to the further increase in the molecular weight of OVA. Xiong et al. (16) reported that the –OH on serine and threonine or the –NH_2_ on lysine and arginine were bound to phosphate groups *via* C–O–P and C–N–P bounds. The addition of phosphate molecules caused steric hindrance that made IgG and IgE more difficult to recognize their linear epitopes of OVA. It is reflected in the reduction of IgG- and IgE-binding capacities of OVA. Moreover, PEF pretreatment could partially unfold the OVA molecule (7), lead to the exposure of phosphorylation sites and finally improve the phosphorylation. Therefore, the IgG- and IgE-binding capacities of phosphorylated OVA were further markedly decreased by the PEF pretreatment.

In order to investigate the possible reason of reduction in the IgG/IgE-binding of OVA induced by phosphorylation combined with PEF pretreatment, the conformational changes of OVA were analyzed by spectrometry. The changes in the content of secondary structure indicated that OVA was unfolded after phosphorylation, and the α-helix content may increase mainly at the expense of β-sheet content. Phosphorylation could make the conformation of protein more flexible at the expense of β-turn (13, 17). Accordingly, the contents of α-helix and unordered structures were increased while that of β-turn was decreased. Besides, phosphorylation could make OVA molecule unfolding and cause exposure of Trp residues, which reflecting in an obvious increase of UV absorbance at 280 nm and a decrease in λ_*max*_ intensity of intrinsic fluorescence that resulted from the quenching of fluorescence intensity (28). In addition, no shift of maximum UV absorbance and a red shift of the maximum emission wavelength were observed, suggesting that phosphorylation partially unfolded OVA structure and made Trp residues exposed on the surface of OVA molecule. Meanwhile, phosphorylation could increase the surface hydrophobicity of OVA through the introduction of phosphate groups *via* covalent bonds. Also, a large number of hydrophobic groups had been exposed due to proteins unfolding (18). The increase of surface hydrophobicity resulted in the harder binding of IgG or IgE with protein (31). The partial unfolding of OVA induced by phosphorylation may result in the damage of conformational IgG and IgE epitopes and lead to the reduction of IgG and IgE-binding of OVA. PEF pretreatment intensified the changes in secondary and tertiary structure of OVA by improving phosphorylation and further reduced the IgG/IgE-binding ability of OVA. Therefore, the destruction of conformational epitopes caused by the phosphorylation combined with PEF pretreatment was another reason for the reduction in IgG- and IgE-binding capacities of OVA.

In addition, the results also showed that dry-heating at 50°C for 2 h had an inconspicuous effect on the IgG- and IgE-binding capacities of OVA, which reflected in the insignificant changes in secondary and tertiary structure of OVA. It may be caused by the movement limitation of OVA molecule because of the low-water activity (4, 32). Moreover, when phosphorylated OVA was pretreated at 30 kV/cm for 180 μs, it exhibited lower IgG- and IgE-binding capacities. This may be the result of aggregation of OVA induced by high-intensity PEF pretreatment (7). The aggregation may lead to the intramolecular and intermolecular hiddenness of some phosphorylation sites of OVA (24). It finally resulted in the increase of IgG- and IgE-binding capacities compared with the phosphorylated OVA without PEF pretreatment.

In conclusion, it was shown that phosphorylation combined with PEF pretreatment dramatically decreased the IgG- and IgE-binding abilities of OVA, which was closely correlated with the structural changes. Moreover, PEF pretreatment promoted the reduction of IgG- and IgE-binding capacities by improving the phosphorylated of OVA. The results imply that PEF pretreatment combined with phosphorylated may be a promising method for OVA desensitization. However, in the future, the phosphorylated sites of OVA treated under the condition should be revealed by mass spectrometry to explore the mechanism of PEF pretreatment combined with phosphorylated reduces the IgG and IgE-binding of OVA at the molecular level. Besides, other experiments *in vivo*, such as skin prick test (SPT) and double-blind placebo-controlled (DBPC) trials, should be implied to prove the decreased allergenicity by PEF pretreatment combined with phosphorylation.

## Data availability statement

The original contributions presented in this study are included in the article/supplementary material, further inquiries can be directed to the corresponding authors.

## Author contributions

WY and QW designed and wrote the manuscript. WY and DD analyzed the data. QL and WD provided the data and revised the manuscript. All authors contributed to the article and approved the submitted version.

## Footnotes

1
http://dichroweb.cryst.bbk.ac.uk/html/process.shtml

## References

[1] Dong X Zhang Y-Q. An insight on egg white: From most common functional food to biomaterial application. *J Biomed Mater Res B Appl Biomater.* (2021) 109:1045–58. 10.1002/jbm.b.34768 33252178

[2] Oriel RC Wang J. Diagnosis and management of food allergy. *Immunol Allergy Clin North Am.* (2021) 41:571–85. 10.1016/j.iac.2021.07.012 34602229

[3] Leech SC Ewan PW Skypala IJ Brathwaite N Erlewyn-Lajeunesse M Heath S BSACI 2021 guideline for the management of egg allergy. *Clin Exp Allergy.* (2021) 51:1262–78. 10.1111/cea.14009 34586690

[4] Yang W Tu Z Wang H Zhang L Song Q. Glycation of ovalbumin after high-intensity ultrasound pretreatment: Effects on conformation, immunoglobulin (Ig)G/IgE binding ability and antioxidant activity. *J Sci Food Agric.* (2018) 98:3767–73. 10.1002/jsfa.8890 29344948

[5] Ma XJ Chen HB Gao JY Hu CQ Li X. Conformation affects the potential allergenicity of ovalbumin after heating and glycation. *Food Addit Contam Part A Chem Anal Control Expo Risk Assess.* (2013) 30:1684–92. 10.1080/19440049.2013.822105 23915026

[6] Seo JH Kim JH Lee JW Yoo YC Kim MR Park KS Ovalbumin modified by gamma irradiation alters its immunological functions and allergic responses. *Int Immunopharmacol.* (2007) 7:464–72. 10.1016/j.intimp.2006.11.012 17321469

[7] Yang W Tu Z Wang H Zhang L Gao Y Li X Immunogenic and structural properties of ovalbumin treated by pulsed electric fields. *Int J Food Prop.* (2017) 20:S3164–76. 10.1080/10942912.2017.1396479

[8] Yang W-H Tu Z-C Wang H Li X Tian M. High-intensity ultrasound enhances the immunoglobulin (Ig)G and IgE binding of ovalbumin. *J Sci Food Agric.* (2017) 97:2714–20. 10.1002/jsfa.8095 27747886

[9] Ma X-J Gao J-Y Chen H-B. Combined effect of glycation and sodium carbonate-bicarbonate buffer concentration on IgG binding, IgE binding and conformation of ovalbumin. *J Sci Food Agric.* (2013) 93:3209–15. 10.1002/jsfa.6157 23553593

[10] Lopez-Exposito I Chicon R Belloque J Recio I Alonso E Lopez-Fandino R. Changes in the ovalbumin proteolysis profile by high pressure and its effect on IgG and IgE binding. *J Agric Food Chem.* (2008) 56:11809–16. 10.1021/jf8023613 19053365

[11] Yang W Tu Z Li Q Kaltashov IA McClements DJ. Utilization of sonication-glycation to improve the functional properties of ovalbumin: A high-resolution mass spectrometry study. *Food Hydrocoll.* (2021) 119:106822. 10.1016/j.foodhyd.2021.106822

[12] Costa J Villa C Verhoeckx K Cirkovic-Velickovic T Schrama D Roncada P Are physicochemical properties shaping the allergenic potency of animal allergens? *Clin Rev Allergy Immunol.* (2022) 62:1–36. 10.1007/s12016-020-08826-1 33411319

[13] Potel CM Kurzawa N Becher I Typas A Mateus A Savitski MM. Impact of phosphorylation on thermal stability of proteins. *Nat Methods.* (2021) 18:757–9. 10.1038/s41592-021-01177-5 34140700

[14] Sánchez-Reséndiz A Rodríguez-Barrientos S Rodríguez-Rodríguez J Barba-Dávila B Serna-Saldívar SO Chuck-Hernández C. Phosphoesterification of soybean and peanut proteins with sodium trimetaphosphate (STMP): Changes in structure to improve functionality for food applications. *Food Chem.* (2018) 260:299–305. 10.1016/j.foodchem.2018.04.009 29699673

[15] Huntington JA Stein PE. Structure and properties of ovalbumin. *J Chromatogr B Biomed Sci Appl.* (2001) 756:189–98. 10.1016/S0378-4347(01)00108-6 11419711

[16] Xiong Z Zhang M Ma M. Emulsifying properties of ovalbumin: Improvement and mechanism by phosphorylation in the presence of sodium tripolyphosphate. *Food Hydrocoll.* (2016) 60:29–37. 10.1016/j.foodhyd.2016.03.007

[17] Sheng L Ye S Han K Zhu G Ma M Cai Z. Consequences of phosphorylation on the structural and foaming properties of ovalbumin under wet-heating conditions. *Food Hydrocoll.* (2019) 91:166–73. 10.1016/j.foodhyd.2019.01.023

[18] Tang S Yu J Lu L Fu X Cai Z. Interfacial and enhanced emulsifying behavior of phosphorylated ovalbumin. *Int J Biol Macromol.* (2019) 131:293–300. 10.1016/j.ijbiomac.2019.03.076 30876897

[19] Xiong Z Ma M. Enhanced ovalbumin stability at oil-water interface by phosphorylation and identification of phosphorylation site using MALDI-TOF mass spectrometry. *Colloids Surf B.* (2017) 153:253–62. 10.1016/j.colsurfb.2017.02.027 28273492

[20] Lv L Chi Y. Improvement of functional properties of ovalbumin phosphorylated by dry-heating in the presence of pyrophosphate. *Eur Food Res Technol.* (2012) 235:981–7. 10.1007/s00217-012-1831-7

[21] Arshad RN Abdul-Malek Z Munir A Buntat Z Ahmad MH Jusoh YMM Electrical systems for pulsed electric field applications in the food industry: An engineering perspective. *Trends Food Sci Technol.* (2020) 104:1–13. 10.1016/j.tifs.2020.07.008

[22] Arshad RN Abdul-Malek Z Roobab U Munir MA Naderipour A Qureshi MI Pulsed electric field: A potential alternative towards a sustainable food processing. *Trends Food Sci Technol.* (2021) 111:43–54. 10.1016/j.tifs.2021.02.041

[23] Yogesh K. Pulsed electric field processing of egg products: A review. *J Food Sci Technol.* (2016) 53:934–45. 10.1007/s13197-015-2061-3 27162373 PMC4837730

[24] Yang W Tu Z Wang H Zhang L Kaltashov IA Zhao Y The mechanism of reduced IgG/IgE-binding of β-lactoglobulin by pulsed electric field pretreatment combined with glycation revealed by ECD/FTICR-MS. *Food Funct.* (2018) 9:417–25. 10.1039/C7FO01082F 29220053

[25] Yang W Tu Z McClements DJ Kaltashov IA. A systematic assessment of structural heterogeneity and IgG/IgE-binding of ovalbumin. *Food Funct.* (2021) 12:8130–40. 10.1039/D0FO02980G 34287434

[26] Zhao W Yang R Lu R Tang Y Zhang W. Investigation of the mechanisms of pulsed electric fields on inactivation of enzyme: Lysozyme. *J Agric Food Chem.* (2007) 55:9850–8. 10.1021/jf072186s 17956144

[27] Yang W Tu Z Wang H Zhang L Xu S Niu C Mechanism of reduction in IgG and IgE binding of β-lactoglobulin induced by ultrasound pretreatment combined with dry-state glycation: A study using conventional spectrometry and high-resolution mass spectrometry. *J Agric Food Chem.* (2017) 65:8018–27. 10.1021/acs.jafc.7b02842 28800703

[28] Zhang Q-T Tu Z-C Xiao H Wang H Huang X-Q Liu G-X Influence of ultrasonic treatment on the structure and emulsifying properties of peanut protein isolate. *Food Bioprod Process.* (2014) 92:30–7. 10.1016/j.fbp.2013.07.006

[29] Zhang Q Tu Z Wang H Huang X Shi Y Sha X Improved glycation after ultrasonic pretreatment revealed by high-performance liquid chromatography–linear ion trap/orbitrap high-resolution mass spectrometry. *J Agric Food Chem.* (2014) 62:2522–30. 10.1021/jf5002765 24606342

[30] Liu J Chen W-M Shao Y-H Zhang J-L Tu Z-C. The mechanism of the reduction in allergenic reactivity of bovine α-lactalbumin induced by glycation, phosphorylation and acetylation. *Food Chem.* (2020) 310:125853. 10.1016/j.foodchem.2019.125853 31757487

[31] Zhang M Tu Z-C Liu J Hu Y-M Wang H Mao J-H The IgE/IgG binding capacity and structural changes of Alaska pollock parvalbumin glycated with different reducing sugars. *J Food Biochem.* (2021) 45:e13539. 10.1111/jfbc.13539 33107047

[32] Matsudomi N Takahashi H Miyata T. Some structural properties of ovalbumin heated at 80 °C in the dry state. *Food Res Int.* (2001) 34:229–35. 10.1016/S0963-9969(00)00157-5

